# Mental health risks and support for frontline workers during the 2013–2016 Ebola outbreak

**DOI:** 10.20935/mhealthwellb7717

**Published:** 2025-05-26

**Authors:** Ira Chaturvedi, Andrew S. Huang, Amy Luo, Alexander H. Chang, J. Lee Jenkins, Edbert B. Hsu

**Affiliations:** 1 Johns Hopkins University, Baltimore, MD 21218, USA.; 2 Department of Population, Family and Reproductive Health, Johns Hopkins Bloomberg School of Public Health, Baltimore, MD 21202, USA.; 3 Lewis Katz School of Medicine, Temple University, Philadelphia, PA 19140, USA.; 4 Department of Emergency Medicine, Johns Hopkins School of Medicine, Baltimore, MD 21287, USA.; 5 Johns Hopkins Office of Critical Event Preparedness and Response, Baltimore, MD 21209, USA.; 6 Johns Hopkins Center for Global Emergency Care, Baltimore, MD 21209, USA.

**Keywords:** Ebola, frontline workers, healthcare workers, mental health, stress, well-being, epidemiology, intervention

## Abstract

The 2013–2016 West African Ebola virus outbreak was the longest and largest Ebola outbreak to date. High levels of stress and isolation experienced by frontline workers (FWs) during the Ebola outbreak highlight the importance of recognizing mental health and well-being (MHW). This study aimed to summarize and synthesize the MHW dimensions and interventions among FWs during the Ebola outbreak. A scoping review was conducted for English- and French-language articles indexed in PubMed and the Global Health Library, published from 2013 up to March 2025. Quantitative and qualitative studies reporting on the epidemiology and interventions for MHW among FWs, including healthcare personnel and ancillary health staff, during the Ebola outbreak were eligible for inclusion. A total of 22 articles were included in this review. Of these, 14 articles addressed the epidemiology of MHW for FWs, 3 addressed MHW interventions for FWs, and 5 addressed both topics. Studies interviewing FWs offered a glimpse into the unique psychological burden of responding to the outbreak, highlighting themes of stress, anxiety, social isolation, fear, and guilt. Intervention-based studies identified resources that could provide psychological support and/or relevant education. Recommendations aimed at the local, national, and international levels are proposed. Studies that address MHW among FWs during the Ebola outbreak are scarce, with even fewer describing interventions that address these issues. Demands on FWs remain an essential consideration during any emergent response, and further examination of impacts and interventions is needed.

## 1. Introduction

Infectious disease outbreaks place markedly increased demands on healthcare systems and personnel. Frontline workers (FWs), including both medical and nonmedical support staff, must deal with challenging work environments, resource constraints, staffing shortages, and increased workloads. In addition, FWs face increased risks of infection through close patient contact and must often adapt to new infection control protocols and unfamiliar practices. For FWs, fears of becoming infected and of the unknown are among the most pressing psychosocial challenges [1]. In responding to infectious outbreaks, FWs experience a daunting combination of stressors that can profoundly affect their mental health and well-being (MHW) [2].

The 2013–2016 West African Ebola virus outbreak was the longest and largest Ebola outbreak to date, as well as the first to spread beyond Central or Eastern Africa [3]. Following the index case in a remote village in Guinea in December 2013, the outbreak spread rapidly throughout the country and to densely populated urban areas in neighboring Sierra Leone and Liberia by mid-2014. During this outbreak, Ebola involved seven more countries in Africa, Europe, and North America. At the end of the outbreak, 28,652 infections and approximately 11,325 deaths in the ten countries were recorded [4]. The adjusted case fatality rates in the three countries at the epicenter of the outbreak rose above 80 percent [5].

FWs were essential to the 2013–2016 West Africa Ebola outbreak response. At the peak of the outbreak, many international organizations sent aid to local healthcare workers in West Africa, with the CDC deploying approximately 1,450 CDC responders to Guinea, Liberia, and Sierra Leone [6]. Additionally, 24,655 medical FWs in West Africa were trained in infection prevention and control practices [7]. Nevertheless, constant exposure from working in Ebola Treatment Units (ETUs) resulted in a 20 to 30 times higher likelihood of infection of FWs compared to the general adult population [8]. Mortality rates among medical FWs were much higher than those of the general population; while mortality rates in Guinea, Sierra Leone, and Liberia were 0.02%, 0.06%, and 0.11%, respectively, medical FW fatality rates were 1.45%, 6.85%, and 8.07%, respectively [9].

As a cornerstone of any infectious outbreak response, FWs will be subject to the short- and long-term mental health impacts of a wide variety of psychological stressors and traumas [10]. Understanding how this impacts the mental health and well-being of FWs is essential to supporting and protecting the workforce [11]. The scope of MHW interventions for FWs during the 2013–2016 West Africa Ebola outbreak has not been comprehensively examined to date. Of note, this may have implications for ways in which the MHW of FWs in other infectious outbreaks in low-resource settings might be addressed. This scoping review aimed to (1) synthesize the epidemiological patterns and key dimensions of MHW and (2) characterize interventions implemented to address MHW among frontline workers during the Ebola outbreak. We contextualize the stressors faced by FWs during the Ebola outbreak and stratify the identified interventions according to local, national, and international levels.

## 2. Materials and methods

We conducted a scoping review of English- and French-language articles indexed in PubMed and the Global Health Library published between December 2013 and 19 March 2025. This period covers the entire span of the 2013–2016 West Africa Ebola outbreak, including its World Health Organization (WHO) declaration as a Public Health Emergency of International Concern (PHEIC) on 8 August 2014.

### 2.1. Search strategy and selection process

Peer-reviewed literature search strategies were developed by the team with a university informationist. The complete search strategies are detailed in Supplementary materials, incorporating PubMed and the Global Health Library for peer-reviewed English- and French-based articles. Duplicates were removed. The results were imported into Covidence (Veritas Health Innovation, Melbourne, Australia), and additional identified duplicates were removed.

During the screening of the title and abstract, articles were required to (1) pertain to FWs; (2) relate to the period during the Ebola outbreak; and (3) include the terms, stress, sleep, mental health, resilience, or wellness. When screening the full text, the inclusion criteria included a description pertaining to epidemiology or intervention or proposed intervention for FWs. Exclusion criteria were applied to articles not describing FW MHW epidemiology or interventions pertaining to FW MHW during the Ebola outbreak. Two members of the team independently assessed each citation. Studies were included only if two reviewers agreed that the studies contained original quantitative or qualitative data addressing one or more of the key questions. In cases of initial disagreement between reviewers regarding the inclusion of a study, it was reviewed by the team to reach a consensus and make a final determination. The inclusion criteria required the article to (1) address one of the key questions. The exclusion criteria were studies (1) not written in English or French; (2) not pertaining to the target population; (3) not related to the topics; (4) not pertaining to the specified time frame; and (5) including no original data (e.g., editorial, commentary, or review articles). For each study that met the inclusion criteria, a team member used Covidence to extract pre-determined information about the characteristics and context of the studies. A second member of the team reviewed the extracted information for accuracy. The extracted information was organized into tables.

### 2.2. Data extraction and synthesis

A PICOTS (population, intervention, comparator, outcomes, timing, setting) typology was developed to guide the scoping review. The population included FWs composed of both medical (e.g., healthcare workers) and nonmedical (e.g., ancillary and support staff) personnel who worked in response to Ebola. Interventions included any interventions or proposed interventions focused on addressing stress and wellness in FWs. Comparators consisted of FWs not working to respond to the Ebola outbreak during the same period. Outcomes included reduced stress or enhanced wellness following an intervention. Timing spanned from 2013 to 2016 during the Ebola outbreak. Setting included any location where FWs encountered those with Ebola virus disease. The findings were narratively synthesized in accordance with the established narrative analysis methods [12].

## 3. Results

### 3.1. Study identification and inclusion

**Figure 1 •:**
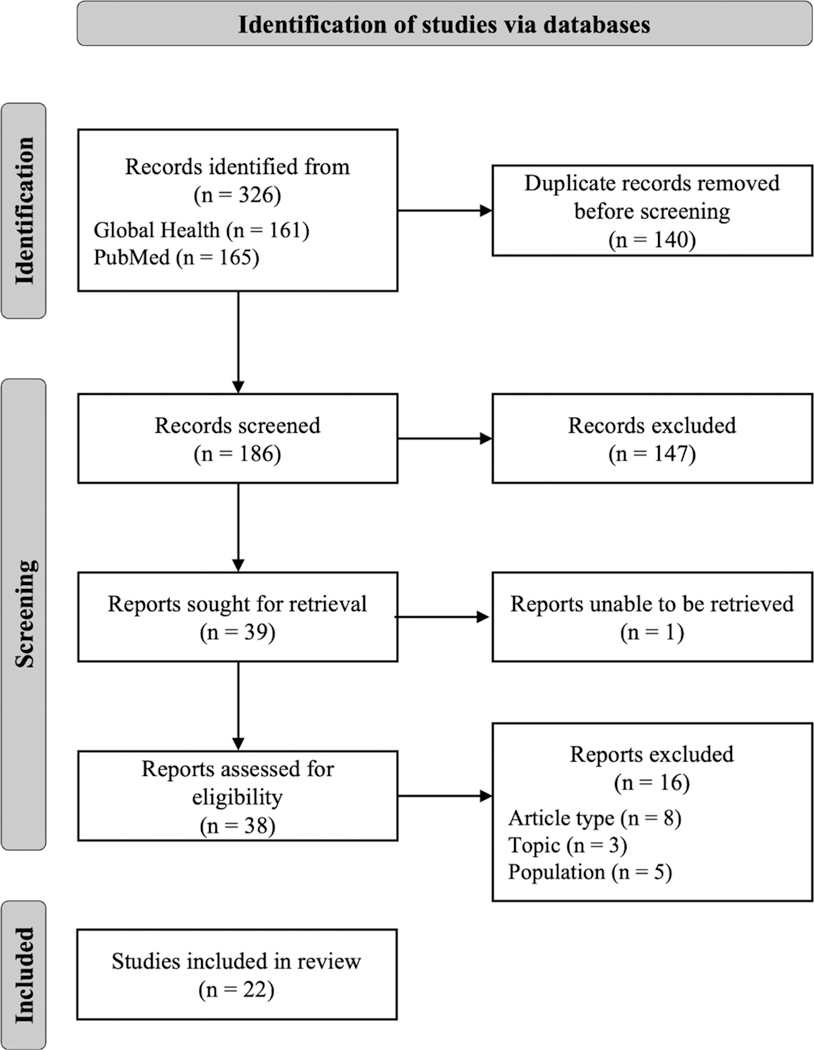
PRISMA-Scoping Review (ScR) diagram.

After removing duplicates, a total of 186 records were retrieved (Figure 1). Following the screening of the title and abstract, we retrieved 39 full-text reports for eligibility. Among these, 1 report was unable to be retrieved and 16 full-text reports were excluded due to wrong article type (e.g., editorial, commentary, or reviews), topic (e.g., unrelated to MHW), or population (e.g., Ebola patients or survivors). The final review included 22 articles.

### 3.2. Characteristics of included studies

The characteristics of the included studies are summarized in Table 1. Among the 22 articles that met the inclusion criteria, 14 (64%) characterized the epidemiology of MHW among FWs in response to the Ebola outbreak, 3 (14%) described interventions implemented to address stress and wellness among FWs, and 5 (23%) included both. Ten (45%) studies were conducted in Sierra Leone, two (9%) studies were conducted in Liberia, one (5%) study was conducted in Guinea, five (23%) studies were conducted in multiple countries in West Africa, two (9%) studies were conducted in Nigeria, two (9%) studies were conducted in Germany, and one (5%) study was conducted in the United States of America (USA). The study populations included local and deployed FWs, including physicians, nurses, medical students, community health workers, auxiliary staff (e.g., hygienists, laboratory technicians), and facility management or leadership members. Among the included studies, 10 (45%) were in-depth interviews, 6 (27%) were cross-sectional, 5 (23%) were case studies, and 2 (9%) were pre-/post-test designs. All studies but one were written in English.

### 3.3. Epidemiological patterns and key dimensions among FWs

Of the twenty-two articles included in this study, nineteen examined the epidemiology of mental health and related morbidities in various FW subpopulations during the 2013–2016 Ebola outbreak [13–30]. These studies used qualitative methods such as interviews or questionnaires to characterize the mental toll on the workforce when responding to the outbreak [15, 21, 22, 24, 25, 27–30]. It was noted that during the Ebola outbreak, mental and psychosocial problems increased, especially in countries at the epicenter of the outbreak, such as Sierra Leone. Some common themes that originated from the literature suggested that FWs felt an increased sense of isolation, moral strain, and psychological distress. Interestingly, while these themes were noted to be present in FWs responding to the Ebola 2013–2016 outbreak, the role of being an HCW was protective to one’s mental health as compared to other Ebola survivors [19, 23].

#### 3.3.1. Social isolation

A recurring theme seen throughout the literature was the sense of isolation that the FWs faced. Compared to medical and research staff not working in ETUs, FWs working in ETUs experienced significantly higher levels of social isolation [13, 15–17, 22, 25, 27, 28, 30]. A factor that increased HCW isolation was the stigma around treating Ebola [13, 15, 27, 28, 30]. Stigma around Ebola extended beyond regional communities at the epicenter of the outbreak, such as Sierra Leone, and was also evident in countries that provided international aid or treated patients, such as the United Kingdom and the United States of America [15, 16, 20, 21, 29, 31, 32]. In addition to community stigma, many FWs feared contagion when working in ETUs, resulting in self-distancing from peers [29, 30]. This fear of contagion extended to households, local communities, and patients, which not only strained and disrupted the interpersonal relationships of FWs but also contributed to feelings of isolation [17, 20, 27, 29, 30].

#### 3.3.2. Moral strain

Another theme was the ethical burden placed on FWs when treating Ebola patients. FWs recall their personal experiences in ETUs and the moral distress that they felt when prioritizing a public health containment approach rather than adopting a patient-centric approach [18, 21]. Moral distress resulted when individuals could not take an action they believed to be ethically correct or were uncertain of what the ethical action was [33]. This distress, in contrast to other forms of distress, threatens one’s core values and has broadly reaching ethical consequences. The alternate standard of care received by patients in ETUs often triggered this distress among FWs during the Ebola outbreak [15].

#### 3.3.3. Psychological distress

The most prevalent theme was the mental health toll faced by FWs in responding to the outbreak. Although various factors can contribute to psychological distress, working in ETUs accounted for significantly higher psychological stress compared to not working in ETUs, with one study showing working in biocontainment units to be more stressful than daily tasks for 60% of respondents [20, 29]. The specific profession of the staff was a determinant of the level of mental burden on FWs. For example, one study showed that medical FWs in Sierra Leone, such as male medics and medical staff who were responsible for cleaning and disinfecting ETUs, had higher scores for OCD, anxiety, phobic anxiety, interpersonal sensitivity, paranoid ideation, and positive symptoms [7]. Positive symptoms accounted for the number of self-reported symptoms of psychopathology respondents experienced. However, this study also noted that FWs with prior experience working in highly stressful medical situations did not view working in ETUs to be as stressful as those lacking similar prior experience, and very serious concerns lessened as measures to address mental health were implemented.

It was also noted that FWs displayed high levels of psychological distress, depression, anxiety, sadness, and prolonged stress [15–17, 22–24, 27, 30]. While there was direct psychological distress due to directly engaging with the Ebola outbreak, FWs also faced higher levels of anxiety, in some cases, due to social and political unrest in the region [17]. Additionally, HCWs also showed increased fear of infection and death, contributing to heightened stress levels in this population [17, 27, 30]. Many FWs also discussed ways in which under-resourced settings were described by some as “a real horror show” [15]. Limited medical resources, PPE, and medical staff demanded long hours for staff, eventually leading to fatigue and depersonalization.

### 3.4. Interventions addressing MHW among FWs

Of the twenty-two articles extracted, eight discussed and/or tested interventions that addressed stress and/or wellness in FWs [14, 27–32, 34]. Many FWs felt that the culmination of various factors previously described led to increased levels of stress and psychological burden, necessitating specific interventions. Strengthening current mental health resources for FWs with the use of mental health specialists to provide requisite psychosocial support or implement cognitive behavioral therapy for FWs was suggested [14, 34]. Studies showed that these mental health resources were used by many of the FWs in ETUs [29]. Studies recommended psychosocial coping methods and other resources for stress management to be offered to FWs [14, 27, 34]. One study incorporated a team of non-specialist nurses who were trained in mental health awareness to support their wards [14]. This training of nurses proved successful in ensuring mental health support for HCWs during the outbreak. Other studies suggested a need for change in the work environment of HCWs, reducing the length of workers’ shifts and placing a larger emphasis on supporting FWs through various services [16]. These interventions should be considered for not only FWs directly working in ETUs but for all FWs. The effectiveness of mobile health (mHealth) interventions in supporting mental health in FWs by helping FWs identify trauma stressors and self-triage was mentioned [32]. Furthermore, in addition to individual-based interventions, studies showed that the inclusion of pre-, peri-, and post-deployment trainings and briefings for international HCWs increased the level of preparedness HCWs felt before working at ETUs and aided in navigating the stressors they faced [25, 31, 32].

## 4. Discussion

This scoping review explored the epidemiology and interventions addressing mental health and wellness pertaining to FWs during the 2013–2016 Ebola outbreak. Exhaustion from long hours in resource-limited settings was commonplace during the outbreak. Local FWs were not the only ones impacted. Many international organizations deployed HCWs who cared for those with EVD, risking their own personal and mental health. Among a total of twenty-two articles, fourteen articles addressed the epidemiology of mental health and wellness among FWs, three articles described interventions for mental health and wellness among FWs, and five articles discussed both epidemiology and interventions in the context of the Ebola outbreak. Along with dealing with workplace stressors, FWs contended with social stressors from their families and their communities. FWs experienced significant social isolation due to the fear and stigma throughout the outbreak to a much greater degree than colleagues who were not in direct contact with Ebola patients. While working in ETUs, many workers dealt with isolation, depression, stigmatization, interpersonal stress, and extreme stress. FWs were required to isolate and quarantine upon leaving the ETU. Additionally, many who worked in ETUs were excluded from social events even after quarantining. The stigma of contracting Ebola continued to impact those at times even following recovery.

These high levels of stress and isolation experienced by FWs working during the Ebola outbreak increased the risks of experiencing mental health issues and led to a high demand for mental health interventions [31, 32, 34, 35]. Although the WHO Mental Gap Action Program (mhGAP) outlined recommendations, countries affected by Ebola lacked mental health and psychosocial support (MHPSS) programs, trained mental health professionals, or the resources to implement these programs [36]. For instance, Liberia and Sierra Leone have a dearth of psychologically trained staff, each with just a single trained psychiatrist, a few dozen mental health nurses, and roughly a thousand trained paraprofessionals trained to assess and manage common mental disorders [23, 37]. The lack of trained personnel amplified the risks of psychological distress and psychopathology [38]. Mental health interventions for the general population were generally implemented with the aid of international organizations, many late into the Ebola outbreak [39, 40].

Several limitations are noted. Although the search strategies were designed to be comprehensive in identifying FWs, including both medical and nonmedical personnel during the Ebola outbreak, other alternate designations of FWs may not have been captured. Studies that did not delineate FW groups were not included given the difficulty of ascertaining the specific impact on FWs. Heterogeneity and limitations of design and outcome measures often limited the comparability of the identified studies. The relative scarcity of the studies which addressed interventions highlights the challenges of conducting intervention-based studies in the most-impacted countries and suggests directions for future research. Recommendations for future mental health interventions encompassing a whole-systems approach at the local, national, and international levels are discussed.

### 4.1. Local-level interventions

Locally, the importance of interventions to directly reduce mental health burdens on FWs cannot be overstated. At the hospital level, one strategy to reduce mental distress of FWs is to decrease shift hours and direct patient interaction time [16, 22]. Various ways to optimize shift loads have been proposed [22]. Balancing shift loads with the increased demand for patient care is a fundamental operational dilemma during outbreaks.

Providing staff with early psychosocial support and strategies for coping with high-stress situations may serve to reduce mental distress among FWs and deploying such interventions as soon as possible is critical [7]. One way to achieve this is through workshops on mental health for FWs. Research shows that the implementation of workshops focused on teaching psychological first aid and coping strategies helps FWs feel more confident not only in being able to access psychosocial support but also in their ability to treat patients [14, 27, 34]. Coping strategies such as using physical protection; practicing self-confidence and pragmatism; and engaging in self-care, peer-support networks, social media platforms, wellness activities, and religion have been shown to reduce mental health burden on FWs [24, 25, 27, 30]. During the COVID-19 pandemic, practicing self-care was an effective intervention for FWs to balance their personal needs with those of their patients [41]. While this requires dedicated time, interventions can be implemented during work shifts, providing FWs with strategies and skills to cope with high levels of stress, burnout, and other mental health issues.

Offering FWs access to psychological support using online and mobile Health (mHealth) platforms that enable them to track their moods, achieve goals, and learn micro-practices can mitigate the psychological burden on FWs [32, 37, 42]. It has been shown that mHealth models allow FWs to tap into a successful self-triage system where they are able to identify traumatic stressors and follow a treatment plan to reduce stress.

**Figure 2 •:**
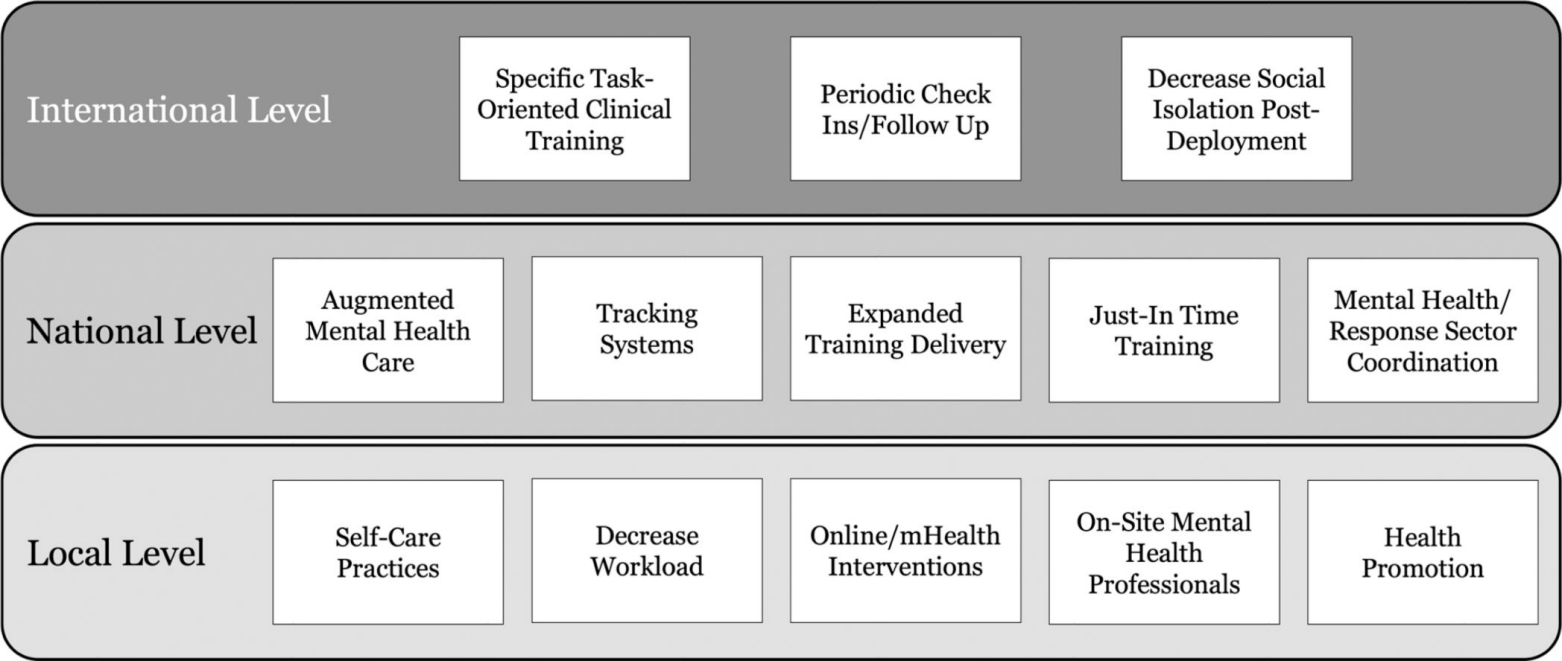
Summary of recommendations.

Incorporating a full-time facility-based mental health practitioner will also reduce FWs’ mental health burden by providing real-time linkages to ongoing psychological care [43]. Though more costly than other interventions, a trained mental health practitioner able to discuss issues privately or in group settings was found to be useful by HCWs. The rapid identification of outbreak-associated psychological stressors, especially for high-risk groups, and the provision of effective coping strategies and resources were also in great demand wherever the 2013–2016 Ebola outbreak occurred. Finally, other types of adjunctive health interventions targeting FWs, such as acupuncture and acupressure, have proven to be successful in reducing anxiety, burnout, and secondary traumatic scores among FWs (Figure 2) [44, 45].

### 4.2. National-level interventions

At the national level, government-sponsored funding and initiatives can also help alleviate the burden of outbreak response on FWs. For example, supplementing the salaries of FWs engaging in outbreak response acknowledges their elevated risk and workload while reducing financial stress [27]. Government agencies must prepare and work with other organizations to ensure that facilities not only have sufficient staff and supplies but also programs and structures in place to foster the mental health of FWs in response to an outbreak. Given the dearth of mental health professionals in regions impacted by the outbreak, efforts should be made to establish additional training programs to develop a skilled deployable cadre [46]. National authorities can also take the lead in the development of systems to track and assist FWs in managing burnout and stress and promote recognition of these impacts on the workforce. Some interventions, such as Community Resiliency Model (CRM) training, focused on body awareness, have proven to help reduce both immediate and long-term stress, with HCWs reporting improved mental well-being as well as decreased stress and somatic symptoms [47]. National organizations can work together to develop similar expanded platforms to provide FWs throughout the country with ready asynchronous access to resources to help cope with high-stress environments. Support at the national level for the development and delivery of training modules on working in stressful and resource-constrained environments could serve as an important preventative measure against burnout.

These measures can be developed and strengthened during normal operations to better address the mental health toll on FWs as part of a comprehensive behavioral health strategy. In the event of an outbreak, wide-scale just-in-time modules specific to the immediate threat, using online and mHealth interventions, may serve as effective, less-costly augmentation measures. As part of any outbreak response planning, the inclusion of mental health professionals pre-, during, and post-outbreak has been strongly recommended [22]. Careful coordination from the mental health and emergency response sectors is essential to identify resources and potential gaps in addressing the mental health needs of FWs to ensure that it is of the highest priority.

### 4.3. International-level interventions

Given the ubiquitous nature of international assistance during outbreak response, systematic approaches to safeguard the mental health of deployed personnel must be taken into consideration. For international medical FWs responding to outbreaks in other countries, the need to improve mental and psychosocial support, especially during pre- and post-deployment, has been well recognized [26]. Including pre-deployment screenings and trainings can help improve response to mental distress by equipping HCWs with stress management tools such as Anticipate, Plan and Deter (APD) training [31, 32]. Additionally, specific task-oriented clinical training during the pre-deployment phase can aid mental preparation for the mission [21, 26]. Including peri-deployment support such as voluntary infield briefings allows HCWs to have the opportunity to share experiences and engage in social networks as well as reduce stress during their deployment [32]. Offering information about Ebola to friends, family, and colleagues back home may help decrease the social isolation of returning participants post-deployment [15, 26]. The post-deployment provision of psychological support to FWs upon their return, such as through periodic check-ins and follow-ups with a mental health specialist, has also been noted to be beneficial [24, 31].

## 5. Conclusions

This scoping review highlights the importance of social isolation, moral strain, and psychological distress impacting FWs as well as a relative scarcity of interventions addressing the mental and social stressors faced during the 2013–2016 Ebola outbreak. The findings suggest a significant demand for mental health intervention development and implementation as part of a comprehensive behavioral health response plan. MWH interventions supporting FWs should be actively explored. Recommendations can be incorporated into healthcare systems during routine operations as well as during outbreak responses to address the gaps in the mental health support of FWs.

## Supplementary Material

Supplemental Materials

Supplementary materials are available at https://doi.org/10.20935/MHealthWellB7717.

## Figures and Tables

**Table 1 •:** Summary of studies on MHW and interventions for FWs during the 2013–2016 Ebola outbreak.

Aim 1: What are the epidemiological patterns and key dimensions of MHW among FWs responding to the Ebola outbreak?							
Title (publication year)	Author [reference]	Study design	FW population	Intervention	Comparators and outcomes	Timing	Setting
The psychological effects of working at an Ebola treatment centre (2017)	Chalk [13]	Case Study	ETU nurses	n/a	Described a “Culture of stigma” experienced by nurses upon exposure and/or contraction of Ebola and underscored the value of mental health support.	2014–2015	Kerry Town, Sierra Leone
Experiences and Psychosocial Impact of West Africa Ebola Deployment on US Health Care Volunteers (2016)	Gershon et al. [15]	In-depth Interviews	11 US deployed physicians; 5 US deployed nurses	n/a	When deployed, HCWs were found to be fearful of exposure, emotionally and physically exhausted, and frustrated with resource limitations, poor management, a lack of clearly defined roles and responsibilities, and an inability to provide high-quality care. Upon returning home, HCWs experienced feelings of isolation, depression, and stigmatization, among others.	2014–2015	West Africa (Guinea, Liberia, and Sierra Leone)
Prevalence of psychological symptoms among Ebola survivors and healthcare workers during the 2014–2015 Ebola outbreak in Sierra Leone: a cross-sectional study (2017)	Ji et al. [19]	Cross-sectional	55 HCWs; 21 logistic staff; 22 medical students; and 41 Chinese deployed medical staff	n/a	Examined obsession–compulsion, anxiety, hostility, phobic anxiety, and paranoid ideation using the Symptoms Checklist 90-items, Revised scale of mental health symptoms. Compared to Ebola survivors, FWs scored lower on all adverse mental health symptoms. FW psychological symptoms declined over 1 month follow-up. Amongst medical staff, Sierra Leone staff had worse mental health symptoms.	2015	Freetown, Sierra Leone
Factors affecting the delivery of healthcare on a humanitarian operation in West Africa: A qualitative study (2018)	Lamb [21]	In-depth Interviews	14 UK Armed Forces medical personnel deployed to West Africa	n/a	In-depth interviews on the challenges of supporting the healthcare mission (e.g., stress, ethical concerns, risk balance, information flow, and pediatric patient distress) and coping behaviors (e.g., confidence in training, leadership, teamwork, peer support, impact).	2014–2015	West Africa
Acute Ebola virus disease patient treatment and health-related quality of life in health care professionals: A controlled study (2016)	Lehmann et al. [20]	Cross-sectional	12 physicians; 26 nurses; 3 laboratory technicians; 1 “other”	n/a	In healthcare professionals, Ebola patient treatment does not seem to be associated with lower health-related quality of life, but it does seem to yield strong(er) feelings of social isolation.	2016	Eppendorf, Germany
Ebola and psychological stress of health care professionals (2015)	Lehmann et al. [16]	Cross-sectional	17 physicians; 29 nurses	n/a	While no significant differences in the severity of somatic symptoms, anxiety, depression, and fatigue emerged between the HCWs who directly contacted Ebola patients and those who did not, those who had direct contact with Ebola patients reported far greater social isolation and felt significantly more need for shorter shift hours.	2014	Eppendorf, Germany
Experiences and challenges in the health protection of medical teams in the Chinese Ebola treatment center, Liberia: a qualitative study (2018)	Li et al. [17]	In-depth Interviews	15 HCWs deployed as part of the People Liberation Army of China medical	n/a	In-depth interviews on the mental health risk facts participants experienced while working in the Ebola Treatment Center. Key themes included disruption of family and social networks, adapting to a different culture, anxiety over social and political unrest in Liberia, and working conditions (supply shortages and long working hours).	2014–2015	Monrovia, Liberia
Mental distress among Liberian medical staff working at the China Ebola Treatment Unit: a cross-sectional study (2015)	Li et al. [7]	Cross-sectional	16 nurses; 36 hygienists	n/a	Mental distress was found to be ‘not very serious’ among local ETU staff due to cooperation and early psychosocial support by the Chinese medical team. Still, it was concluded that study data implied that the psychological health of medical staff within special environments such as this should warrant further attention and/or investigation.	2015	Monrovia, Liberia
‘There was no good choice’: An ethics case study from the Ebola response (2015)	McKay [18]	Case Study	n/a	n/a	Barriers to mental health and well-being included a lack of adequate testing, lack of PPE/supplies, and a disconnected healthcare system.	2015	“A country in West Africa”
Healthcare providers on the frontlines: a qualitative investigation of the social and emotional impact of delivering health services during Sierra Leone’s Ebola epidemic (2016)	McMahon et al. [22]	In-depth Interviews	11 nurses; 8 community health workers; 9 maternal health aides; 1 laboratory technicians, 5 “other”	n/a	FWs described how the Ebola outbreak weakened trust within and across health facilities, providers, communities, and households, which led to strained relationships among providers and between providers and patients. Providers themselves also reported a profound sense of stigmatization, suffering, loneliness, isolation, and sadness since the beginning of the outbreak. To address these, health systems must work to improve mental health and psychosocial support for providers across the board, whether working in designated Ebola treatment and care facilities or not.	2014–2015	Sierra Leone
An evaluation of psychological distress and social support of survivors and contacts of Ebola virus disease infection and their relatives in Lagos, Nigeria: a cross-sectional study–2014 (2015)	Mohammed et al. [35]	Cross-sectional	45 HCWs	n/a	Survivors of Ebola, their contacts, and their relations were found to develop notable psychological distress, warranting greater MH interventions.	2014	Lagos, Nigeria
Nurses in an Ebola Viral Hemorrhagic Fever Outbreak: Facing and Preparing for Psychosocial Challenges (2020)	Paillard-Borg et al. [24]	In-depth Interviews	44 International Federation of Red Cross and Red Crescent deployed nurses	n/a	In-depth interviews described the psychosocial aspects pre-, during, and post-deployment. Key factors that improved MHW among participants included stress management and coping strategies (self-confidence, pragmatism, wellness activities, and peer support) and workplace characteristics (positive professional working environment, trust in the organization). Factors that led to a mixed impact on MHW were during the post-deployment period, where stress was often higher, and other’s attitudes and receptions to participants upon return ranged between pariahs and heroes.	2014–2015	Kenema, Sierra Leone
What adaptation to research is needed following crises: a comparative, qualitative study of the health workforce in Sierra Leone and Nepal (2018)	Raven et al. [25]	In-depth Interviews	6 HCWs; 5 managers; 3 Health Facility Management Committee members	n/a	In-depth interviews revealed that FWs reported feeling stigmatized by the Ebola outbreak that led to a breakdown of trust between treatment and non-treatment facilities and between health authorities and community members. Coping strategies included finding renewed dedication to serve their communities, peer and family support, and their religion.	2011–2014	Sierra Leone
Global nursing in an Ebola viral haemorrhagic fever outbreak: before, during and after deployment (2017)	von Strauss et al. [26]	Cross-sectional	44 nurses	n/a	The following areas were identified for improvement in order to lessen the burden on HCWs (specifically nurses): increased mental health and psychosocial support, pre- and post-deployment coping strategies, more pre-deployment task-oriented clinical training, and workload reduction.	2014–2015	Kenema, Sierra Leone
Aim 2: What interventions were implemented to address MHW among frontline workers during the Ebola outbreak?							
Title	Author(s)	Study design	FW population	Intervention	Comparators/outcomes	Timing	Setting
CDC’s Multiple Approaches to Safeguard the Health, Safety, and Resilience of Ebola Responders (2020)	Klomp et al. [31]	Case Study	US Centers for Disease Control and Prevention public health professionals deployed to West Africa	Pre-deployment screening, resiliency training, and briefing (e.g., peer support, coping skills, stress management, triage, and proper referral process, symptoms of stress, self-care and social support); in-field voluntary operational briefings; post-deployment outreach (e.g., individual and group non-clinical conversations reflecting on deployment experience).	Pre- and post-resiliency training knowledge, self-efficacy, overview of the course content, and training effectiveness.	2009–2016	West Africa
Maximizing the Resilience of Healthcare Workers in Multi-Hazard Events: Lessons from the 2014–2015 Ebola Response in Africa (2019)	Schreiber et al. [32]	Case Study	US medical effort; Ebola HCWs	Pre-deployment Anticipate, Plan and Deter (APD) training for FW pre-deployment. PsychSTART-Responder (PsychSTART-R) self-triage system, a web-based application to identify traumatic stress risk factors and prompt use of resilience plan developed during APD training.	Case study on the implementation of the APD and PsychSTART-R interventions.	2014–2015	West Africa
Training peers to treat Ebola centre workers with anxiety and depression in Sierra Leone (2018)	Waterman et al. [34]	Pre/Post-Test	3273 ETU staff members	Cognitive behavioral therapy interventions, including workshops on psychological first aid, psycho-education on mental health difficulties and coping strategies (e.g., stress, sleep, anxiety, relationship, and behavioral change), and group CBT sessions.	Based on pre- and post-intervention design, the intervention was effective in improving a variety of MHW measures including stress, sleep, post-traumatic stress disorder, anxiety, depression, behavioral and relationship problems, and anger.	2015–2016	Sierra Leone
Both Aim 1 and Aim 2							
Title	Author(s)	Study design	FW population	Intervention	Comparators/outcomes	Timing	Setting
Mental health care during the Ebola virus disease outbreak in Sierra Leone (2017)	Kamara et al. [14]	Case Study	>100 physicians, nurses, and auxiliary staff	Mental health workshops on coping with stigma, discrimination, stress management, and self-care. One-on-one counseling to participants requiring more support.	n/a	2015–2016	Freetown, Sierra Leone
Health workers’ experiences of coping with the Ebola epidemic in Sierra Leone’s health system: a qualitative study (2018)	Raven et al. [27]	In-depth Interviews	25 HCWs; 19 members of the District Health Management Team, heath facility managers, or international partners	Ebola response FWs that were helpful in addressing FW MHW included supplemental salary during the outbreak and training and workshops that strengthened FWs’ confidence in their patient care and psychosocial support.	In-depth interviews on the challenges faced by health workers and coping strategies. Challenges included the breakdown of trust between community members and healthcare workers, isolation from families, fear of infection, trauma from watching colleagues die, stress, and workload. Coping strategies included drawing upon one’s religion, sense of serving one’s country and community, peer and family support, social media platforms, workshops to deal with stigma, and supplements to health worker salary.	2015	Sierra Leone
Ebola in Guinea: Experiences of Stigma among Health Professional Survivors [French title: Ebola en Guinée: formes de la stigmatisation des acteurs de santé survivants] (2016)	Sow et al. [28]	In-depth Interviews	7 physicians; 5 technical health officers; 4 nurses; 2 biologists; 1 laboratory technician; 1 midwife– all of whom were Ebola survivors	Ebola survivor networks and group forums addressing psychosocial needs; Government and NGO financial and food assistance	In-depth interviews described the stigma, outcomes, and coping strategies experienced by Ebola-surviving FWs. Stigma appeared to be rooted in fear and misconceptions of Ebola infection and spread. Stigma resulted in rejection in their own healthcare facility by colleagues. Coping strategies included support groups with other survivors, including FWs.	2015	Conakry, Guinea
The Psychosocial Challenges of Caring for Patients with Ebola Virus Disease (2017)	Smith et al. [29]	In-depth Interviews	3 physicians; 8 nurses; 10 support staff	Behavioral health counseling	Mental health was described as “an important supportive service” and, as expected, counseling and support services were used often. It was also found that working in the biocontainment unit was more stressful than everyday work for 60% of respondents, underscoring the need for support.	2014	Nebraska, USA
How do health workers experience and cope with shocks? Learning from four fragile and conflict-affected health systems in Uganda, Sierra Leone, Zimbabwe and Cambodia (2017)	Witter et al. [30]	In-depth Interviews	5 physicians; 1 nurse; 10 nurses; 3 community health workers; 6 midwives; 1 laboratory technician	Community-based and donor-assisted food, material (personal protective equipment), and financial assistance	Found that HCWs experienced fear of death, fear of patients, changed family dynamics, community stigma, increased stress, lack of supplies, and economic challenges. Some coping strategies were described, including using greater physical protection, social media platforms, and increased intrapersonal interactions (which offered encouragement and support to HCWs).	2015	Sierra Leone

MHW: mental health and well-being; FW: frontline worker; ETU: Ebola Treatment Unit; HCW: healthcare worker; US: United States of America; UK: United Kingdom.

## Data Availability

All data supporting the findings of this publication are available within this article and its Supplementary Materials.

## References

[1] Cabarkapa S, Nadjidai SE, Murgier J, Ng CH. The psychological impact of COVID-19 and other viral epidemics on frontline healthcare workers and ways to address it: a rapid systematic review. Brain Behav Immun-Health. 2020;8:100144. doi: 10.1016/j.bbih.2020.100144 32959031 PMC7494453

[2] Xiong Y, Peng L. Focusing on health-care providers’ experiences in the COVID-19 crisis. Lancet Glob Health. 2020; 8:e740–1. doi: 10.1016/S2214-109X(20)30214-X 32573442 PMC7190304

[3] Oleribe OO, Salako BL, Ka MM, Akpalu A, McConnochie M, Foster M, Ebola virus disease epidemic in West Africa: lessons learned and issues arising from West African countries. Clin Med. 2015;15:54–7. doi: 10.7861/clinmedicine.15-1-54 PMC4954525 25650199

[4] Forna A, Nouvellet P, Dorigatti I, Donnelly CA. Case fatality ratio estimates for the 2013–2016 West African ebola epidemic: application of boosted regression trees for imputation. Clin Infect Dis. 2020;70:2476–83. doi: 10.1093/cid/ciz678 31328221 PMC7286386

[5] WHO Ebola Response Team. Ebola virus disease in West Africa—the first 9 months of the epidemic and forward projections. N Engl J Med. 2014;371:1481–95. doi: 10.1056/NE-JMoa1411100 25244186 PMC4235004

[6] Dahl BA. CDC’s response to the 2014–2016 ebola epidemic— guinea, liberia, and sierra leone. MMWR Suppl. 2016;65:12–20. doi: 10.15585/mmwr.su6503a3 27388930

[7] Li L, Wan C, Ding R, Liu Y, Chen J, Wu Z, Mental distress among Liberian medical staff working at the China Ebola Treatment Unit: a cross sectional study. Health Qual Life Outcomes. 2015;13:156. doi: 10.1186/s12955-015-0341-2 26409446 PMC4583730

[8] World Health Organization. Health worker Ebola infections in Guinea, Liberia and Sierra Leone: a preliminary report. Geneva: WHO; 2015.

[9] Evans DK, Goldstein M, Popova A. Health-care worker mortality and the legacy of the Ebola epidemic. Lancet Glob Health. 2015;3:e439–40. doi: 10.1016/S2214-109X(15)00065-0 26163833

[10] Hill JE, Harris C, Danielle LC, Boland P, Doherty AJ, Benedetto V, The prevalence of mental health conditions in healthcare workers during and after a pandemic: systematic review and meta-analysis. J Adv Nurs. 2022;78: 1551–73. doi: 10.1111/jan.15175 35150151 PMC9111784

[11] Greenberg N, Wessely S, Wykes T. Potential mental health consequences for workers in the Ebola regions of West Africa–a lesson for all challenging environments. J Ment Health. 2015;24:1–3. doi: 10.3109/09638237.2014.1000676 25587816

[12] Popay J, Roberts H, Sowden A, Petticrew M, Arai L, Rodgers M, Guidance on the conduct of narrative synthesis in systematic reviews: a product from the ESRC Methods Programme. Lancaster: Lancaster University; 2006. doi: 10.13140/2.1.1018.4643

[13] Chalk M. The psychological effects of working at an Ebola treatment centre. Br J Nurs Mark Allen Publ. 2017;26:178–9. doi: 10.12968/bjon.2017.26.3.178 28185489

[14] Kamara S, Walder A, Duncan J, Kabbedijk A, Hughes P, Muana A. Mental health care during the Ebola virus disease outbreak in Sierra Leone. Bull World Health Organ. 2017;95:842–7. doi: 10.2471/BLT.16.190470 29200525 PMC5710077

[15] Gershon R, Dernehl LA, Nwankwo E, Zhi Q, Qureshi K. Experiences and psychosocial impact of West Africa Ebola Deployment on US Health Care Volunteers. PLoS Curr. 2016;21;8. doi: 10.1371/currents.outbreaks.c7afaae124e35d2da39ee7e07291b6b5 PMC5074701 27803840

[16] Lehmann M, Bruenahl CA, Löwe B, Addo MM, Schmiedel S, Lohse AW. Ebola and psychological stress of Health Care Professionals. Emerg Infect Dis. 2015;21:913–4. doi: 10.3201/eid2105.141988 25897490 PMC4412243

[17] Li Y, Wang H, Jin X-R, Li X, Pender M, Song C-P, Experiences and challenges in the health protection of medical teams in the Chinese Ebola treatment center, Liberia: a qualitative study. Infect Dis Poverty. 2018;7:92. doi: 10.1186/s40249-018-0468-6 30134982 PMC6103862

[18] McKay G. ‘There was no good choice’: an ethics case study from the Ebola response. Nurs Ethics. 2015;22:827–30. doi: 10.1177/0969733015593858 26338281

[19] Ji D, Ji Y-J, Duan X-Z, Li W-G, Sun Z-Q, Song X-A, Prevalence of psychological symptoms among Ebola survivors and healthcare workers during the 2014–2015 Ebola outbreak in Sierra Leone: a cross-sectional study. Oncotarget. 2017;8:12784–91. doi: 10.18632/oncotarget.14498 PMC5355054 28061463

[20] Lehmann M, Bruenahl CA, Addo MM, Becker S, Schmiedel S, Lohse AW, Acute Ebola virus disease patient treatment and health-related quality of life in health care professionals: a controlled study. J Psychosom Res. 2016;83:69–74. doi: 10.1016/j.jpsychores.2015.09.002 26423938

[21] Lamb D. Factors affecting the delivery of healthcare on a humanitarian operation in West Africa: a qualitative study. Appl Nurs Res. 2018;40:129–36. doi: 10.1016/j.apnr.2018.01.009 29579487

[22] McMahon SA, Ho LS, Brown H, Miller L, Ansumana R, Kennedy CE. Healthcare providers on the frontlines: a qualitative investigation of the social and emotional impact of delivering health services during Sierra Leone’s Ebola epidemic. Health Policy Plan. 2016;31:1232–9. doi: 10.1093/heapol/czw055 27277598 PMC5035780

[23] Mohammed A, Sheikh TL, Poggensee G, Nguku P, Olayinka A, Ohuabunwo C, Mental health in emergency response: lessons from Ebola. Lancet Psychiatry. 2015;2:955–7. doi: 10.1016/S2215-0366(15)00451-4 26544738

[24] Paillard-Borg S, Holmgren J, Saaristo P, Von Strauss E. Nurses in an ebola viral hemorrhagic fever outbreak: facing and preparing for psychosocial challenges. Sage Open. 2020;10:2158244020920658. doi: 10.1177/2158244020920658

[25] Raven J, Baral S, Wurie H, Witter S, Samai M, Paudel P, What adaptation to research is needed following crises: a comparative, qualitative study of the health workforce in Sierra Leone and Nepal. Health Res Policy Syst. 2018;16:6. doi: 10.1186/s12961-018-0285-1 29415738 PMC5804047

[26] Von Strauss E, Paillard-Borg S, Holmgren J, Saaristo P. Global nursing in an Ebola viral haemorrhagic fever outbreak: before, during and after deployment. Glob Health Action. 2017;10:1371427. doi: 10.1080/16549716.2017.1371427 29017025 PMC5645654

[27] Raven J, Wurie H, Witter S. Health workers’ experiences of coping with the Ebola epidemic in Sierra Leone’s health system: a qualitative study. BMC Health Serv Res. 2018;18:251. doi: 10.1186/s12913-018-3072-3 29622025 PMC5887191

[28] Sow S, Desclaux A, Taverne B, PostEboGui Groupe d’étude. Ebola en Guinée: formes de la stigmatisation des acteurs de santé survivants. Bull Société Pathol Exot. 2016;109:309–13. doi: 10.1007/s13149-016-0510-5 27456158

[29] Smith MW, Smith PW, Kratochvil CJ, Schwedhelm S. The psychosocial challenges of caring for patients with ebola virus disease. Health Secur. 2017;15:104–9. doi: 10.1089/hs.2016.0068 28192056

[30] Witter S, Wurie H, Chandiwana P, Namakula J, So S, Alonso-Garbayo A. How do health workers experience and cope with shocks? Learning from four fragile and conflict-affected health systems in Uganda, Sierra Leone, Zimbabwe and Cambodia. Health Policy Plan. 2017;32:3–13. doi: 10.1093/heapol/czx112 29149313

[31] Klomp RW, Jones L, Watanabe E, Thompson WW. CDC’s multiple approaches to safeguard the health, safety, and resilience of ebola responders. Prehospital Disaster Med. 2020;35:69–75. doi: 10.1017/S1049023X19005144 31818341 PMC7113416

[32] Schreiber M, Cates DS, Formanski S, King M. Maximizing the resilience of healthcare workers in multi-hazard events: lessons from the 2014–2015 ebola response in Africa. Mil Med. 2019;184:114–20. doi: 10.1093/milmed/usy400 30901435

[33] Jameton A. Dilemmas of moral distress: moral responsibility and nursing practice. AWHONNs Clin Issues Perinat Womens Health Nurs. 1993;4:542–51. 8220368

[34] Waterman S, Hunter E, Cole CL, Evans LJ, Greenberg N, Rubin GJ. Training peers to treat Ebola centre workers with anxiety and depression in Sierra Leone. Int J Soc Psychiatry. 2018;64:156–65. doi: 10.1177/0020764017752021 29432085

[35] Mohammed A, Sheikh TL, Gidado S, Poggensee G, Nguku P, Olayinka A, An evaluation of psychological distress and social support of survivors and contacts of Ebola virus disease infection and their relatives in Lagos, Nigeria: a cross sectional study−2014. BMC Public Health. 2015;15:824. doi: 10.1186/s12889-015-2167-6 26307047 PMC4550041

[36] World Health Organization. Psychological first aid during Ebola virus disease outbreaks. Geneva: World Health Organization; 2014.

[37] Kabir KS, Flis A, Mickens M, Trapp SK, Wiese J, Lewy H, “We’re not meant to deal with crisis for a year”: supporting frontline healthcare providers’ wellness during a pandemic. Pervasive Computing Technologies for Healthcare. Berlin/Heidelberg: Springer International Publishing; 2022.

[38] Shultz JM, Baingana F, Neria Y. The 2014 Ebola Outbreak and Mental Health: Current Status and Recommended Response. JAMA. 2015;313:567–8. doi: 10.1001/jama.2014.17934 25532102

[39] Cénat JM, Mukunzi JN, Noorishad P-G, Rousseau C, Derivois D, Bukaka J. A systematic review of mental health programs among populations affected by the Ebola virus disease. J Psychosom Res. 2020;131:109966. doi: 10.1016/j.jpsychores.2020.109966 32087433

[40] Hughes P. Mental illness and health in Sierra Leone affected by Ebola: lessons for health workers. Intervention. 2015;13:60–9. doi: 10.1097/WTF.0000000000000082

[41] Søvold LE, Naslund JA, Kousoulis AA, Saxena S, Qoronfleh MW, Grobler C, Prioritizing the mental health and well-being of healthcare workers: an urgent global public health priority. Front Public Health. 2021;9:679397. doi: 10.3389/fpubh.2021.679397 34026720 PMC8137852

[42] Yoon S, Goh H, Nadarajan GD, Sung S, Teo I, Lee J. Perceptions of mobile health apps and features to support psychosocial well-being among frontline health care workers involved in the COVID-19 pandemic response: qualitative study. J Med Internet Res. 2021;23:e26282. doi: 10.2196/26282 33979296 PMC8168635

[43] Inter-Agency Standing Committee (IASC). IASC guidelines on mental health and psychosocial support in emergency settings. Geneva: IASC; 2007. 10.1080/09540261.2022.2147420 36502397

[44] Afrasiabi J, McCarty R, Hayakawa J, Barrows J, Lee K, Plouffe N, Effects of acupuncture and acupressure on burnout in health care workers: a randomized trial. J Trauma Nurs. 2021;28:350–62. doi: 10.1097/JTN.0000000000000614 34766929

[45] Reilly PM, Buchanan TM, Vafides C, Breakey S, Dykes P. Auricular acupuncture to relieve health care workers’ stress and anxiety: impact on caring. Dimens Crit Care Nurs. 2014; 33:151–9. doi: 10.1097/DCC.0000000000000039 24704740

[46] Wirsiy FS, Tahmo NB, Tatah L, Brett-Major DM. Resilience of mental health services amidst Ebola disease outbreaks in Africa. Front Public Health. 2024;12:1369306. doi: 10.3389/fpubh.2024.1369306 38873302 PMC11169587

[47] Grabbe L, Higgins MK, Baird M, Pfeiffer KM. Impact of a resiliency training to support the mental well-being of frontline workers: brief report of a quasi-experimental study of the community resiliency model. Med Care. 2021;59:616–21. doi: 10.1097/MLR.0000000000001535 33827106 PMC8191373

