# Supplementary material for: Mental health risks and support for frontline workers during the 2013–2016 Ebola outbreak
Source: Acad Ment Health Well Being. Author manuscript; Available in PMC 2026 Feb 28. (PMC12948306; doi:10.20935/mhealthwellb7717)
Supplement: Supplemental Materials [file NIHMS2122620-supplement-Supplemental_Materials.pdf]

ACADEMIA
MENTAL HEALTH &
WELL-BEING

Supplementary Materials

Search Strategies for PubMed and Global Health

PubMed search:

("Hemorrhagic Fever, Ebola"[Mesh] OR "Ebola"[tw] OR "ebolavirus"[tw]) AND ("health personnel"[mh:noexp] OR "allied health personnel"[mh:noexp] OR "emergency medical technicians"[mh] OR "paramedics"[mh] OR "Anesthetists"[Mesh] OR "anesthesiologists"[mh] OR "nurse anesthetists"[mh] OR "infection control practitioners"[mh] OR "medical staff, hospital"[mh] OR "hospitalists"[mh] OR "nurses"[mh] OR "nursing staff"[mh] OR "personnel, hospital"[mh:noexp] OR "nursing staff, hospital"[mh] OR "physicians"[mh:noexp] OR "general practitioners"[mh] OR "pediatricians"[mh] OR "physicians, family"[mh] OR "physicians, primary care"[mh] OR "pulmonologists"[mh] OR "pulmonologist*"[tw] OR "health personnel"[tw] OR "healthcare personnel"[tw] OR "health care personnel"[tw] OR "healthcare provider*"[tw] OR "health care provider*"[tw] OR "healthcare worker*"[tw] OR "health care worker*"[tw] OR "healthcare professional*"[tw] OR "health care professional*"[tw] OR "allied health professional*"[tw] OR "emergency medical technician*"[tw] OR "EMT"[tw] OR "EMTs"[tw] OR "paramedic*"[tw] OR "anesthetist*"[tw] OR "anesthesiologist*"[tw] OR "infection control practitioners"[mh] OR "infection control practitioner*"[tw] OR "infection control physician*"[tw] OR "hospital medical staff"[tw] OR "physician*"[tw] OR "hospital registrar*"[tw] OR "hospitalist*"[tw] OR "nurse"[tw] OR "nurses"[tw] OR "nursing staff"[tw] OR "nursing personnel"[tw] OR "physician*"[tw] OR "doctor*"[tw] OR "general practitioner*"[tw] OR "pediatrician*"[tw] OR "primary care"[tw] OR "pulmonologist*"[tw] OR "clinical officer*" OR "pharmacists"[mh] OR "pharmacist*"[tw] OR "pharmacy technicians"[mh] OR "pharmacy technician*"[tw] OR "psychologist*"[tw] OR "community health workers"[mh] OR "community health worker*"[tw] OR "community health assistant*"[tw] OR "community health officer*"[tw] OR "community mental health aide*"[tw] OR "dental assistants"[mh] OR "dental assistant*"[tw] OR "dental nursing auxiliary"[tw] OR "dentists"[mh] OR "dentist*"[tw] OR "oral and maxillofacial surgeons"[mh] OR "oral surgeon*"[tw] OR "endodontists"[mh] OR "endodontist*" OR "dental surgeon*"[tw] OR "dental technician*"[tw] OR "dental therapist*"[tw] OR "dental hygienist*"[tw] OR "district health officer*"[tw] OR "ebola holding unit*"[tw] OR "ebola treatment unit*"[tw] OR "ebola treatment center*"[tw] OR "environmental health officer*"[tw] OR "epidemiologists"[mh] OR "epidemiologist*"[tw] OR "surgeons"[mh] OR "general surgeon*"[tw] OR "health officer*"[tw] OR "internist*"[tw] OR "laboratory personnel"[mh] OR "medical laboratory personnel"[mh] OR "laboratory assistant*"[tw] OR "laboratory technician*"[tw] OR "maternal-child health services"[mh] OR "maternal and child health"[tw] OR "maternal child health"[tw] OR "medical laboratory scientific officer*"[tw] OR "medical laboratory science officer*"[tw] OR "medical

laboratory officer*"[tw] OR "medical officer*"[tw] OR "chief health officer*"[tw] OR "midwife"[tw] OR "midwives"[tw] OR "birthing attendant*"[tw] OR "nursing assistants"[mh] OR "nursing assistant*"[tw] OR "nursing aide*"[tw] OR "nursing officer*"[tw] OR "nutritionists"[mh] OR "nutritionist*"[tw] OR "obstetricians"[mh] OR "obstetrician*"[tw] OR "gynecologists"[mh] OR "gynecologist*"[tw] OR "occupational therapists"[mh] OR "occupational therapist*"[tw] OR "ophthalmologists"[mh] OR "ophthalmologist*"[tw] OR "optician*"[tw] OR "optometrists"[mh] OR "optometrist*"[tw] OR "outbreak investigation"[tw] OR "pathologists"[mh] OR "pathologist*"[tw] OR "pediatricians"[mh] OR "pediatrician*"[tw] OR "pediatric assistants"[mh] OR "pediatric assistant*"[tw] OR "physical therapists"[mh] OR "physical therapist*"[tw] OR "physical therapist assistants"[mh] OR "physical therapist assistant*"[tw] OR "physical therapy assistant*"[tw] OR "psychiatrist*"[tw] OR "public health aide*"[tw] OR "public health assistant*"[tw] OR "public health officer*"[tw] OR "radiology assistant*"[tw] OR "radiology technician"[tw] OR "hospital rapid response team"[mh] OR "rapid response"[tw] OR "respiratory technician"[tw] OR "respiratory therapist"[tw] OR "medical receptionists"[mh] OR "receptionist*"[tw] OR "dental auxiliaries"[mh] OR "maintenance and engineering, hospital"[mh] OR "hospital maintenance"[tw] OR "hospital manager*"[tw] OR "hospital secretar*"[tw] OR "laundry service, hospital"[mh] OR "hospital laundry service"[tw] OR "launderer"[tw] OR "hospital logistics"[tw] OR "logistics officer*"[tw] OR "medical equipment technician*"[tw] OR "medical records department, hospital"[mh] OR "medical records department"[tw] OR "medical records assistant*"[tw] OR "medical records officer*"[tw] OR "social workers"[mh] OR "social worker*"[tw] OR "case managers"[mh] OR "case manager*"[tw] OR "mortician*"[tw] OR "mortuary attendant*"[tw] OR "burial team*"[tw] OR "burial staff"[tw] OR "pastoral care"[mh] OR "pastoral care"[tw] OR "registration clerk*"[tw] OR "rehabilitation worker*"[tw] OR "rehabilitation assistant*"[tw] OR "security service*"[tw] OR "security officer*"[tw] OR "security worker*"[tw])
AND
("occupational stress"[mh] OR "burnout, professional"[mh] OR "stress, psychological"[mh:noexp] OR "burnout, psychological"[mh] OR "psychological well-being"[mh] OR "resilience, psychological"[mh] OR "mental health"[mh] OR "occupational stress*"[tw] OR "job stress*"[tw] OR "work related stress*"[tw] OR "work place stress*"[tw] OR "professional stress*"[tw] OR "job related stress*"[tw] OR "burnout"[tw] OR "burn out"[tw] OR "psychological stress*"[tw] OR "well-being"[tw] OR "wellness"[tw] OR "resilience"[tw] OR "resilient"[tw] OR "mental health"[tw])
AND 2013:2017[dp] AND eng[la]
Searched 10/11/2023, 39 results

Global Health Library:

(exp ebola haemorrhagic fever/ OR "Ebola".mp OR "ebolavirus".mp) AND

(exp health care workers/ OR exp medical auxiliaries/ OR exp hospital personnel/ OR exp nurses/ OR exp physicians/ OR exp general practitioners/ OR exp pediatricians/ OR "pulmonologist*".mp OR "health personnel".mp OR "healthcare personnel".mp OR "health care personnel".mp OR "healthcare provider*".mp OR "health care provider*".mp OR "healthcare worker*".mp OR "health care worker*".mp OR "healthcare professional*".mp OR "health care professional*".mp OR "allied health professional*".mp OR "emergency medical technician*".mp OR "EMT".mp OR "EMTs".mp OR "paramedic*".mp OR "anesthetist*".mp OR "anesthesiologist*".mp OR "infection control practitioner*".mp OR "infection control physician*".mp OR "hospital medical staff".mp OR "physician*".mp OR "hospital registrar*".mp OR "hospitalist*".mp OR "nurse".mp OR "nurses".mp OR "nursing staff".mp OR "nursing personnel".mp OR "physician*".mp OR "doctor*".mp OR "general practitioner*".mp OR "pediatrician*".mp OR "primary care".mp OR "pulmonologist*".mp OR "clinical officer*" OR exp pharmacists/ OR "pharmacist*".mp OR "pharmacy technician*".mp OR "psychologist*".mp OR exp community health workers/ OR "community health worker*".mp OR "community health assistant*".mp OR "community health officer*".mp OR "community mental health aide*".mp OR "dental assistant*".mp OR "dental nursing auxiliary".mp OR exp dentists OR "dentist*".mp OR "oral surgeon*".mp OR "endodontist*" OR "dental surgeon*".mp OR "dental technician*".mp OR "dental therapist*".mp OR "dental hygienist*".mp OR "district health officer*".mp OR "ebola holding unit*".mp OR "ebola treatment unit*".mp OR "ebola treatment center*".mp OR "environmental health officer*".mp OR "epidemiologist*".mp OR exp surgeons/ OR "general surgeon*".mp OR "health officer*".mp OR "internist*".mp OR exp laboratory workers/ OR "laboratory assistant*".mp OR "laboratory technician*".mp OR "maternal and child health".mp OR "maternal child health".mp OR "medical laboratory scientific officer*".mp OR "medical laboratory science officer*".mp OR "medical laboratory officer*".mp OR "medical officer*".mp OR "chief health officer*".mp OR exp midwives/ OR "midwife".mp OR "midwives".mp OR "birthing attendant*".mp OR "nursing assistant*".mp OR "nursing aide*".mp OR "nursing officer*".mp OR exp nutritionists/ OR "nutritionist*".mp OR "obstetrician*".mp OR "gynecologist*".mp OR "occupational therapist*".mp OR "ophthalmologist*".mp OR "optician*".mp OR "optometrist*".mp OR "outbreak investigation".mp OR "pathologist*".mp OR "pediatrician*".mp OR "pediatric assistant*".mp OR "physical therapist*".mp OR "physical therapist assistant*".mp OR "physical therapy assistant*".mp OR "psychiatrist*".mp OR "public health aide*".mp OR "public health assistant*".mp OR "public health officer*".mp OR "radiology assistant*".mp OR "radiology technician".mp OR "rapid response".mp OR "respiratory technician".mp OR "respiratory therapist".mp OR "receptionist*".mp OR "hospital maintenance".mp OR "hospital manager*".mp OR "hospital secretar*".mp OR "hospital laundry service".mp OR "launderer".mp OR "hospital logistics".mp OR "logistics officer*".mp OR "medical equipment technician*".mp

OR "medical records department".mp OR "medical records assistant*".mp OR "medical records officer*".mp OR exp social workers/ OR "social worker*".mp OR "case manager*".mp OR "mortician*".mp OR "mortuary attendant*".mp OR "burial team*".mp OR "burial staff".mp OR "pastoral care".mp OR "registration clerk*".mp OR "rehabilitation worker*".mp OR "rehabilitation assistant*".mp OR "security service*".mp OR "security officer*".mp OR "security worker*".mp)
AND
(exp burnout/ OR exp mental stress/ OR exp mental health/ OR "occupational stress*".mp OR "job stress*".mp OR "work related stress*".mp OR "work place stress*".mp OR "professional stress*".mp OR "job related stress*".mp OR "burnout".mp OR "burn out".mp OR "psychological stress*".mp OR "well-being".mp OR "wellness".mp OR "resilience".mp OR "resilient".mp OR "mental health".mp)

Additional staff terms:

Medical staff

Anesthesiologist, anesthetist, assistant anesthetist, clinical officer, clinical pharmacist, clinical psychologist, community health assistant, community health officer, community health officer, community mental health aide, critical care nurse, dental nursing auxiliary/assistant, dental surgeon, dentist, dental technician, dental therapist/hygienist/nurse, district health officer, ebola holding unit, ebola treatment unit, ebola treatment center, environmental health officer, epidemiologist, family physician, general surgeon, health officer, infection control, infectious disease, internal medicine, internist, laboratory assistant, laboratory technician, maternal and child health aide, medical laboratory scientific officer, medical officer, mental health CHO, mental health nurses, midwife (SECHN), nurse, nursing aide, nursing officer, nutritionist, obstetrics, gynecology, birthing attendant, occupational therapy, ophthalmologist, optician, optometrist, outbreak investigation, pathologist, pediatrician, pediatric nurse, pharmacist, pharmacy technician, physical therapy, physician, psychologist, psychiatrist, pulmonologist, public health aide, public health officer, radiology assistant, rapid response, respiratory technician, respiratory therapist, senior nursing officer, community health worker, nurse, nurse midwife, radiology technician

Nonmedical staff

Receptionist, hospital maintenance staff, team, hospital manager, hospital secretary, launderer, logistics officer, medical equipment technician, medical records assistant, medical records officer, mental health, social worker, case manager, mortician, mortuary attendant, burial team, burial staff, pastoral care, registration clerk, rehabilitation worker, security.
